# An Interactive Web-Based Platform for Support Generation and Optimisation for Metal Laser Powder Bed Fusion

**DOI:** 10.3390/ma17071639

**Published:** 2024-04-03

**Authors:** Antonios Dimopoulos, Giorgos Chryssinas, Dimitra Mavroforaki, Tat-Hean Gan, Panagiotis Chatzakos

**Affiliations:** 1 Department of Mechanical and Aerospace Engineering, Brunel University London, Uxbridge UB8 3PH, UK; tat-hean.gan@brunel.ac.uk; 2 TWI Hellas, Leof. Kifisias 280, 152 32 Chalandri, Greece; george.chryssinas@twi.gr (G.C.); dimitra.mavroforaki@twi.gr (D.M.); panagiotis.chatzakos@twi.gr (P.C.); 3 TWI Ltd., Granta Park, Great Abington, Cambridge CB21 6AL, UK

**Keywords:** additive manufacturing, metal support structures, metal laser powder bed fusion, support generation, support optimisation, web platform, PySLM library, python programming

## Abstract

Powder bed fusion—laser beam (PBF-LB), a prevalent and rapidly advancing additive manufacturing (AM) technology nowadays, serves the industry by producing thin, complex, and lightweight components for various sectors, including healthcare, automotive, defence, and aerospace. However, this technology encounters challenges related to the construction of critical parts and the high overall process costs. Equally significant is the role of support structures in metal laser powder bed fusion (PBF-LB/M). The absence of supports can lead to defective and collapsed parts, while the incorrect selection of a support type or the addition of unnecessary supports results in increased material usage and additional post-processing efforts. Therefore, there is a pressing need for advanced software capable of generating appropriate support structures and predicting the thermomechanical behaviour of a part under PBF-LB/M printing conditions. Such software would be beneficial for the industry to avoid printing defects caused by high thermal stresses, minimise material usage, reduce printing time, and ensure high-quality prints. In this study, we introduce a web-based support generation and optimisation platform for PBF-LB/M. Through this platform, among other features, users can import three-dimensional (3D) parts and generate block-type support structures with diamond perforations based on the PySLM library, all within a user-friendly web environment. The first release of the platform (v0.6) is fully interactive and accessible online at no cost.

## 1. Introduction

The ISO/ASTM terminology standard defines additive manufacturing as the overarching term for technologies that incrementally fuse material, typically layer by layer, to fabricate physical objects according to 3D model data specifications. These technologies are presently used for various applications in the engineering industry as well as other areas of society, such as medicine, education, architecture, cartography, toys and entertainment [1]. Due to the reduced time required to make prototypes, these technologies are also referred to as “rapid prototyping”, and the prospect of employing them as a manufacturing process has become highly appealing [2]. According to the ISO/ASTM classification, there are seven major categories of AM technologies: powder bed fusion (PBF), directed energy deposition (DED), material jetting (MJT), binder jetting (BJT), material extrusion (MEX), vat photopolymerisation (VPP), and sheet lamination (SHL) [1]. They are primarily classified based on their processing mechanisms, with each category further divided into various processes depending on the energy source and the materials used. Metals and polymers (including photopolymers) are extensively utilised materials in AM.

Metal laser powder bed fusion, also known as selective laser melting (SLM) is classified as a powder bed fusion—laser beam (PBF-LB) additive technology according to the ISO/ASTM standards [1]. It is among the frequently utilised and swiftly advancing additive manufacturing technologies and can be directly used for the precise and high-performance fabrication of metal components across various industries, including aerospace, biomedical, defence, and automotive [3,4]. In PBF-LB/M, a laser beam selectively melts powder within a powder bed, and numerous melting tracks are subsequently fused together in a micro-welding process. This process consolidates multiple layers to form a 3D component within the powder envelope [5]. A broad range of materials, such as aluminium, nickel, and titanium, can be utilised in PBF-LB/M. The mechanical properties of the resulting parts can be similar to or surpass those produced through conventional methods like machining and moulding [3]. Moreover, compared to other metal-based AM technologies, PBF-LB/M systems generate fully dense parts directly from the process, and thus no additional thermal or chemical procedures are required to solidify the material [6].

Nevertheless, numerous challenges emerge for this technology concerning the creation of overhang structures and the distortion caused by thermal stresses [7]. In PBF-LB/M, support structures are always required, since the unmelted powder surrounding the component cannot provide support for the overhanging surfaces and alleviate thermal stresses, as Poyraz et al. [8] and Bo and Chou [9] found in their studies. During the solidification process, the material in the build undergoes multiple cycles of melting and cooling, accumulating stress due to uneven heating. This residual stress poses significant issues, as it can lead to problems such as warping, cracks, and part delamination [10,11]. The support structures serve multiple functions, including anchoring the part on the build plate, providing a stable platform for the next layer to be built upon, and acting as a heat sink for controlled cooling. However, their selection requires careful consideration. Producing an object without or with insufficient supports can lead to distorted and collapsed parts. On the other hand, incorporating excessive supports or selecting an inappropriate support type amplifies post-processing requirements, elongates the time and effort necessary for their removal, increases the risk of part damage, and augments the amount of material needed [12]. Hence, over the last few years, there has been growing interest from the industry and researchers in developing optimised support structures that enhance the efficiency of the printing process for PBF-LB/M systems.

Considerable research has been undertaken in recent years to advance the design, generation, and optimisation of metal support structures aiming to enhance the efficiency of the printing process and ensure the production of flawless objects with zero defects. Several of these studies focus on optimising support structures through testing and experimentation, as emphasised in the literature section of the authors’ previously published work [13]. The novel design methods proposed aim to create support structures that are easily removable and utilise minimal material while maintaining the quality of the printed part. Other studies concentrate on examining the thermal expansion and deformation behaviour of parts during the printing process for various materials, utilising numerical modelling and computer-aided thermomechanical simulations. The proposed approaches aim to predict and evaluate the thermomechanical behaviour of metal parts when exposed to high temperatures or PBF-LB/M printing conditions. This is directed towards the fabrication of optimised metal support structures capable of addressing these conditions and thereby eliminating defects such as warping, distortion, cracks, and part delamination. These studies are highlighted in the literature section of another author’s earlier published work [14], where the thermal behaviour of four common support structures used in metal AM was investigated. The aim was to propose optimal parameters that minimise support volume and residual stress while ensuring high-quality printed outcomes.

Many commercial software options are available for support structure design and generation, allowing users to modify and tune support parameters and generate support geometries according to the imported model. Materialise Magics [15] is one of the most commonly used software packages for support design and generation in PBF-LB/M. It allows users to set part orientation, select support geometry from a range of types including block, line, point, web, contour, gusset, combi, volume, and cones, generate support structures automatically or manually based on the imported model, and modify and tune the various support parameters. Enhanced iterations of Materialise Magics include a powerful tool known as Materialise e-Stage, a fully automatic support generation solution developed to bridge the experience gap and reduce data preparation time, powder consumption, and post-processing. The software packages Amphyon [16] and 3DXpert [17] developed by Oqton (San Francisco, CA, USA, https://oqton.com/, accessed on 25 January 2024) are simulation-based process preparation tools for metal AM providing new analysis and simulation features capable of supporting every stage of the AM workflow, from design to printing. With this software, users can maximise part performance through topology optimisation, minimise manufacturing costs for reduced printing time and material consumption, and ensure thermal stability and non-defective parts by performing thermal and structure simulations calculating residual stress and distortions. AM-Studio software (https://www.cads-additive.com/en/am-studio-data-processing-software-3d-printing/, accessed on 25 January 2024) [18], developed by Hexagon and CADS Additive, is another high-end AM solution that guides the user through the entire printing process covering every step, including post-processing. It allows users to define part orientation, automatically repair damaged 3D files, and generate innovative support geometries such as tree support and AdaptiveCell support in addition to the traditional block, rod and line. 4D_Additive Manufacturing Software Suite (https://www.coretechnologie.com/products/4d-additive.html, accessed on 25 January 2024) [19] by Core Technologies can read 3D models from all common CAD (computer-aided design) formats, transforming them directly into an exact, intelligent, light B-Rep geometry suitable for AM processes. Through the support generation module, users can specify the optimal part orientation, and a wide variety of special support structures can be generated in automatic and/or manual mode. One more powerful 3D printing build preparation software compatible not only with metal but also with plastic AM technologies is VoxelDance Additive (https://voxeldance.com/voxeldance_additive, accessed on 25 January 2024) [20]. In addition to repairing 3D files, establishing optimal part orientation, and efficient nesting, the support generation module offers a diverse selection of support geometries, including bar, point, line, block, and smart supports. Autodesk Fusion 360 with Netfabb [21] offers a complete toolset for design and implementation in AM. Within this platform, users can import 3D files, analyse printability, repair models, generate support structures and reduce build failures via process simulation that calculates residual stresses and part distortion.

However, the majority of the aforementioned support generation tools rely on geometry and predefined patterns, lacking the ability to position support structures based on the thermal stresses encountered in PBF-LB/M. As a result, creating support structures demands extensive knowledge of additive manufacturing and frequently becomes an iterative process, largely guided by the user’s printing experience with analogous geometries. The majority of the published research explores the optimisation of support structures, focusing solely on geometric design and experimental outcomes without considering the constraints of thermal stress and deformation. Meanwhile, support optimisation has been investigated in few studies, taking into consideration multiple aspects simultaneously, including part orientation, geometric support parameters, and thermal stresses applied in PBF-LB/M.

In this research, for the first time, an initial release of an interactive web-based platform for support generation and optimisation is proposed. The platform allows users to import 3D files, determine part orientation, generate and visualise block-type support structures with diamond perforations using the open-source PySLM library developed by Luke Parry [22], access optimisation suggestions for block, line, contour, and cone supports based on the author’s prior research [14], perform thermomechanical simulations to anticipate the thermal performance of the imported part, as well as export the prepared assembly for further processing. The platform targets not only professionals but also intermediate users involved in metal AM, providing user-friendly functionality within a simple interface. The first release (v0.6) is fully interactive and accessible online at no cost.

## 2. Methodology

### 2.1. Web Architecture and Modules

The web application leverages the React.js frontend framework. This is essentially a JavaScript library to build user interfaces, and for a particular project stands out against other frameworks and libraries. React’s distinctive feature is its virtual Document Object Model (DOM), a virtual representation of the actual DOM tree. This virtual DOM renders swiftly, a crucial attribute of the application, particularly given its high demand and the complexity of handling 3D objects on the web. React’s component-based logic is a standout feature, contributing to cleaner, more robust, and concise code across the entire application. This is not a conventional web application: it necessitates web-based visualisation of 3D models. Typically, most web-based 3D applications rely on the Three.js library, an open-source JavaScript library that encapsulates WebGL, providing a user-friendly application programming interface (API) for creating and displaying animated 3D computer graphics. The interaction of React and Three.js shows that there are ways to combine those two technologies to create a React application with 3D dynamic. React-three-fibre builds dynamic scene graphs declaratively with reusable components, which makes dealing with Three.js easier and comes to terms with the component-oriented React logic. These components dynamically respond to state changes and facilitate user interaction.

**Figure 1:**
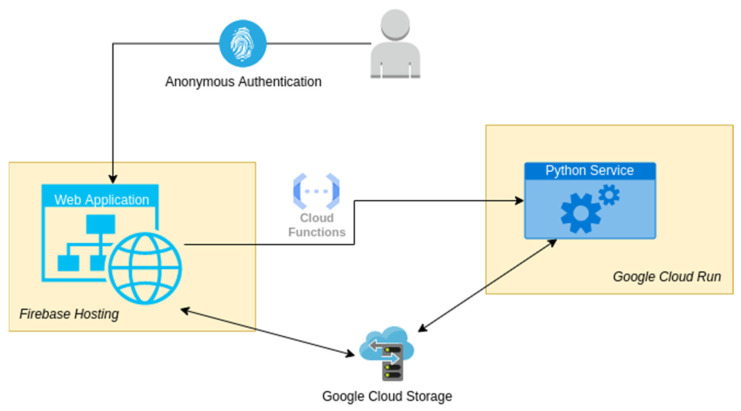
Web application and Python service connection scheme.

The major functionality of the application is gathered in a web platform and a Python microservice. The web platform exploits the capabilities of Firebase version 13.6.0. Firebase (https://firebase.google.com/, accessed on 10 February 2024) is a software development platform acquired by Google. The Python microservice is hosted on a Google Virtual Machine (VM) server. During the development of the project, the need to integrate the two computing services (the backend Python microservice, resting on a Google Cloud VM, and the frontend Firebase service) has appeared. A unified Google Cloud–Firebase project gives the user the flexibility to upload and download files via the Firebase Software Development Kits (SDKs) (client side), in addition to potential server-side processing using the Google Cloud Storage API (https://cloud.google.com/, accessed on 10 February 2024). Specifically, the connection between the web application and the Python service is established through a Representational State Transfer Application Programming Interface (REST API) and shared cloud storage, as shown in the scheme of Figure 1. Employing shared cloud storage eliminates the necessity of transferring large binary files via hypertext transfer protocol (HTTP) requests. It is reasonable to anticipate that deploying the Web Application and the Python Service in the same data-centre region results in considerably faster direct file Input/Output compared to the process of sending and receiving files through HTTP requests. Integrating the existing Firebase platform with the Google Cloud project not only automates file handling but also simplifies the current architecture, reducing both the required cost and implementation time.

As previously noted, among the platform’s features, users import 3D files, orient the component, generate support structures, access support optimisation suggestions, perform thermomechanical simulations, and eventually export the finalised job, all within a user-friendly web environment. The first release of the platform is equipped with three main modules: the support generation module, the support optimisation module, and the thermomechanical simulation module. A comprehensive, step-by-step description of the modules along with their various features is presented in the following sections, accompanied by technical characteristics derived from the platform’s development.

### 2.2. Import and Orientation

The platform can read various geometries in STL (stereolithography), a format that is most commonly used in AM to represent the surface of the CAD model using triangular meshes. Once the part is imported into the platform, the overhang surfaces where support structures are required are automatically highlighted in red. The part’s orientation is also automatically generated upon import, providing the user with a range of options to choose from, allowing them to select the one that best satisfies the criteria for achieving optimal printability. At present, the suggested orientations are listed randomly; however, in future iterations, the optimal orientation will be prioritised at the top, determined by minimising the required support material, printing time, and overall costs. Furthermore, the “surface angle” feature enables the definition of the minimum angle at which support structures will be generated. Regarding the scene’s manipulating views, “rotate view” can be achieved through the right mouse button, “zoom in/out view” through the mouse scroll wheel, and “pan view” through the middle mouse button.

From a technical perspective, as noted above, a variety of suggested orientations are generated for the imported part. Some of these orientations are generated as orthocanonical rotations based on the initial orientation, while others are determined based on the geometric characteristics of the part to eliminate overhang surfaces. The orientation calculations and most of the geometric manipulations are performed using Trimesh [23], a Python library for 3D mesh manipulation.

### 2.3. Support Generation Module

**Figure 2:**
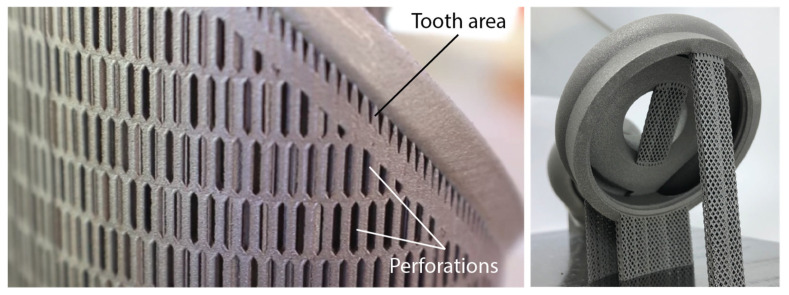
Block-type support structures with diamond perforations.

Through the support generation module, block-type support structures with diamond perforations are created beneath the overhang surfaces highlighted in red. These support structures are widely employed in PBF-LB/M and SLM 3D printers due to their capacity to enhance the printability of complex parts, eliminate significant defects like warping, and reduce both build time and powder consumption (less trapped powder), all while maintaining high standards for post-processing activities such as support removal. Their morphology [24,25] is characterised by vertical thin walls arranged in a grid pattern, diamond perforations on each wall, and a tooth area representing the contact area between the part and the supports, as illustrated in Figure 2. Using the platform’s generation engine, various support parameters can be modified and tuned, resulting in the final geometry of the supports. For instance, “x, y, hatching” is a parameter of block-type support structures determined by the spacing between vertical walls. “Beam” and “angle” are two parameters that define the shape and the frequency of the diamond-shaped pattern, while “tooth height” and “tooth top length” parameters indicate the geometry of the tooth area. Among the various parameters of this support type, which can be found in many commercial slicers, the hatching distance and the diamond pattern have been exclusively investigated and developed in this first iteration. The tooth area, along with other relevant parameters that define the geometry of the supports, will be explored in future iterations.

The support generation is accomplished by utilising the PySLM library [22], an open-source library that offers utilities (such as slicing, hatching, and support generation) for SLM and direct metal laser sintering (DMLS) AM processes. PySLM is a relatively new library, still in the development phase. However, its support generation functionality suffices for our initial experimentation.

### 2.4. Support Optimisation Module

The support optimisation module is an interactive engine that provides optimisation suggestions for commonly used geometries in metal AM and PBF-LB/M systems, including block, line, contour, and cones support structures. The optimisation results are based on the authors’ previously published work [14], where the thermal behaviour of the above-mentioned support types was studied aiming to minimise support volume and residual stress while maintaining the quality of prints. Specifically, support density and tooth area were examined using various support parameters, including x, y hatching, tooth height, and tooth top length, while design of experiments (DOE) methodology was employed to create support alternatives. Both the alternatives and the small ledge specimens used were thermomechanically analysed to propose solutions that optimise support volume, thermal stress, supports’ heat transmission, and overhang displacement. Thus, this optimisation engine enables users to modify and fine-tune input support parameters for each type through user-friendly horizontal sliders, all while simultaneously displaying the corresponding support optimisation levels.

The software Design Expert 13 [26] was utilised to prepare the DOE and create the alternatives for each support type. Additionally, based on analysis of variance (ANOVA) results, the respective mathematical models were employed in the same software to perform and visualise the optimisation outcome. A representative example of the models used is illustrated by Equation (1), demonstrating the volume of block-type support structures. The complete set of equations used to develop the support optimisation module is accessible in the Supplementary Materials.

Support Volume = 6991.79410 − 137.65809 × Tooth Height + 137.90833 × Tooth Top Length − 5247.51284 × X, Y Hatching + 71.08333 × Tooth Height × Tooth Top Length + 43.15556 × Tooth Height × X, Y Hatching − 117.00000 × Tooth Top Length × X, Y Hatching − 0.004938 × Tooth Height^2^ − 0.277778 × Tooth Top Length^2^ + 1281.04691 × X, Y Hatching^2^. (1)

### 2.5. Simulation Module

The simulation module enables users to perform thermomechanical simulations on both the imported model and the generated support structures, providing information on thermal stress, temperature, and displacement within an interactive and user-friendly web interface. This feature is designed to evaluate the thermal behaviour of a print job under PBF-LB/M printing conditions. Its goal is to assist users in predicting potential issues that might result in defective or collapsed parts, ensuring parallel optimal printability. In this first release, the simulation module operates in demo mode, offering functionality exclusively for a selected ledge specimen. The simulation scene, including the specimen, the meshing, and the generated supports, was first prepared in COMSOL Multiphysics software (version 6.1, Burlington, MA, USA) [27]. The material used was grade 4 titanium, a strong and lightweight Ti alloy commonly employed in PBF-LB/M, while a value of 1550 °C (usually the melting point of Ti alloys used in AM) was set under the overhang surface, representing the heat source. The simulation data, including mesh, thermal stress, temperature, and displacement, were exported from COMSOL and then imported into the web optimisation platform to visualise the numerical results.

Hence, from a technical standpoint, a coloured mesh is generated by integrating the mesh used in COMSOL for the simulation and incorporating the simulation results. Once again, the Trimesh Python library is employed to create and export the coloured mesh. In future iterations, a network will be established that enables effective communication between the simulation software and the web platform through shared online servers. This will allow users to set up and conduct thermomechanical simulations directly within the web platform, also utilising their preferred 3D model.

### 2.6. Solid Supports and Export

As previously mentioned, the platform generates support structures using the PySLM library. These structures are characterised by non-solid geometries, making the exploration of solid supports a particularly challenging aspect for our web platform. First, generating solid support structures (or converting non-solid ones into solid) is a more convenient way to visualise the supports in relation to the imported part. Solid supports are also essential for conducting and visualising numerical simulations, as non-solid geometries are unreadable for any type of simulation software, given that mesh generation is not feasible. Finally, the thickness of the support structures must be defined before exporting the final build job to the machine and proceeding with the printing process. Our platform allows users to convert the initially generated non-solid supports into solid ones, while also providing the option to modify the thickness values. Moreover, the final assembly, including both the imported part and the supports, can be exported in STL format and utilised for further analysis or slicing preparation before the final print. Future iterations will focus on improving the communication between the web platform and 3D printing machines’ compatibility, enabling users to efficiently prepare and export a finalised build job that can be printed without the need for additional slicing processing.

To generate thick support structures from the original 2D support structures, the Madcad Python library [28] was employed. This library is capable of conducting all the necessary calculations to extrude the 2D shape of the supports into a 3D shape with the desired thickness. In the 2D supports, problematic triangles may exist, which need to be filtered out. This is achieved by examining characteristics such as the triangles’ area and normal vectors.

## 3. Implementation and Discussion

### 3.1. User Journey Overview

**Figure 3:**
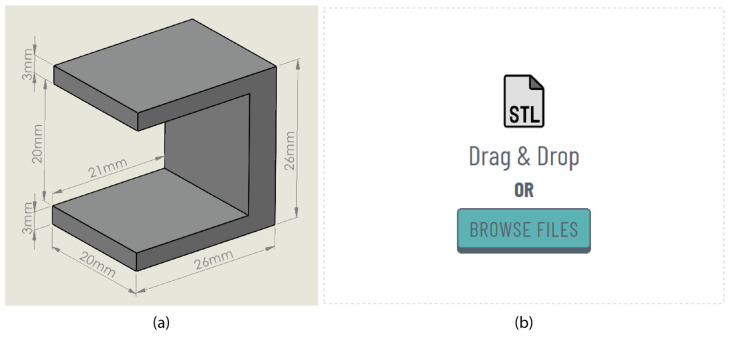
(**a**) Ledge specimen; (**b**) “Import” panel.

To gain a deeper insight into the functionality of the proposed web optimisation platform based on the features described above, this section provides a detailed user journey utilising a small ledge specimen. This specimen is available in the Supplementary Materials titled ‘STL specimen_1’, with its dimensions illustrated in Figure 3a. The initial step involves importing a 3D model in STL format, as shown in Figure 3b. Users can accomplish this by either dragging and dropping the preferred file or selecting a file from their local file system.

**Figure 4:**
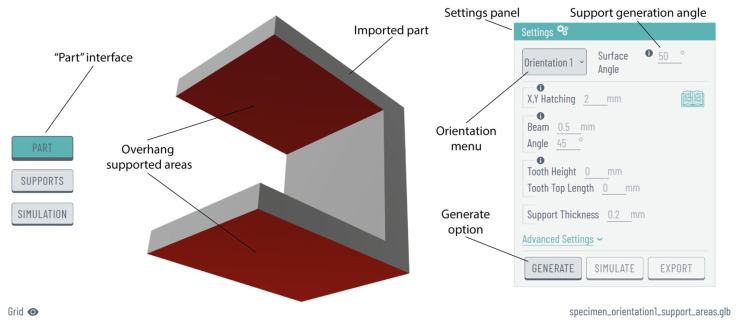
“Part” interface and commands after importing the part into the platform.

After importing the part, users can choose their desired orientation from a variety of options presented in the “orientation” drop-down menu. Once the support generation angle is specified (the minimum angle to generate supports), the platform identifies and highlights in red the critical overhang areas where supports are required. This process is illustrated in Figure 4, where the imported model, the highlighted areas, and the primary “settings” panel are displayed within the “part” interface.

**Figure 5:**
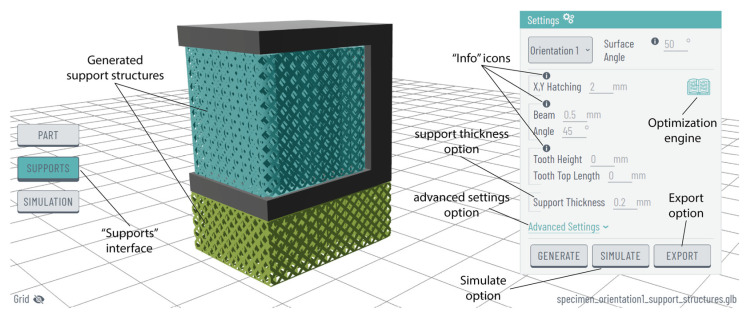
“Supports” interface, generated support structures, and commands.

**Figure 6:**
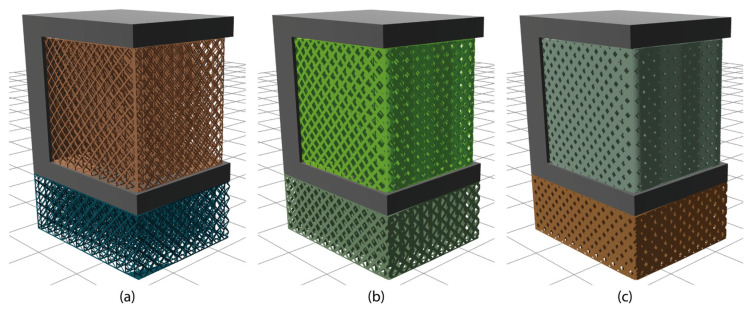
Three support samples of different beam values: (**a**) x, y hatching = 2, beam = 0.2; (**b**) x, y hatching = 2, beam = 0.4; (**c**) x, y hatching = 2, beam = 0.8.

**Figure 7:**
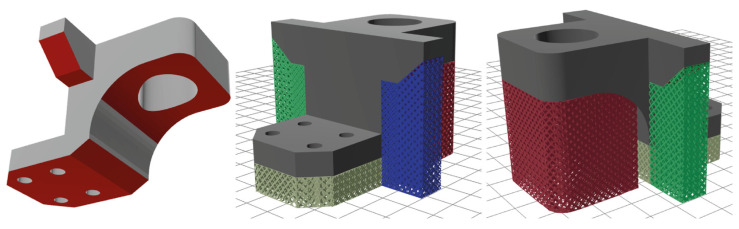
Support generation process on a custom bear bracket model.

By choosing the “generate” option, and moving forward to the “supports” interface, the platform creates block-type support structures with diamond perforations based on the PySLM library as shown in Figure 5. In the “settings” panel, default values have been assigned to parameters such as x, y hatching, beam, angle, tooth height, and tooth top length; however, the platform provides users the flexibility to customise these values accordingly. Furthermore, an “advanced settings” option is available for users seeking greater expertise in AM. This option provides additional support parameters, including support border distance, projection resolution, inner/outer support edge gap, triangulation spacing and more. To exemplify, Figure 6 illustrates three samples of support structures generated by using the same ledge specimen, each employing different support parameters. Here, it should be noted that in each part, all support domains were configured with the same settings. However, future iterations will feature domain selection, enabling the application of different support settings for each support domain. Additionally, to further showcase the platform’s capability in metal support generation, a more complex part, designated as ‘STL specimen_2’ in the Supplementary Materials, was incorporated, as illustrated in Figure 7.

**Figure 8:**
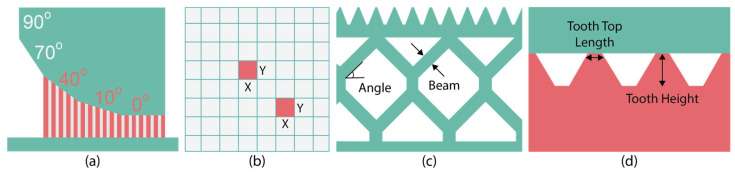
(**a**) Generation angle; (**b**) x, y hatching; (**c**) beam, angle; (**d**) tooth height, tooth top length.

Moreover, the “information” icons positioned above each parameter (Figure 5) serve as a valuable guide, providing users with detailed information panels, including content and figures, related to the respective support parameter. For instance, Figure 8a provides a graphical representation of the support generation angle, Figure 8b of x, y hatching, Figure 8c of beam and angle, and Figure 8d of tooth height and tooth top length.

**Figure 9:**
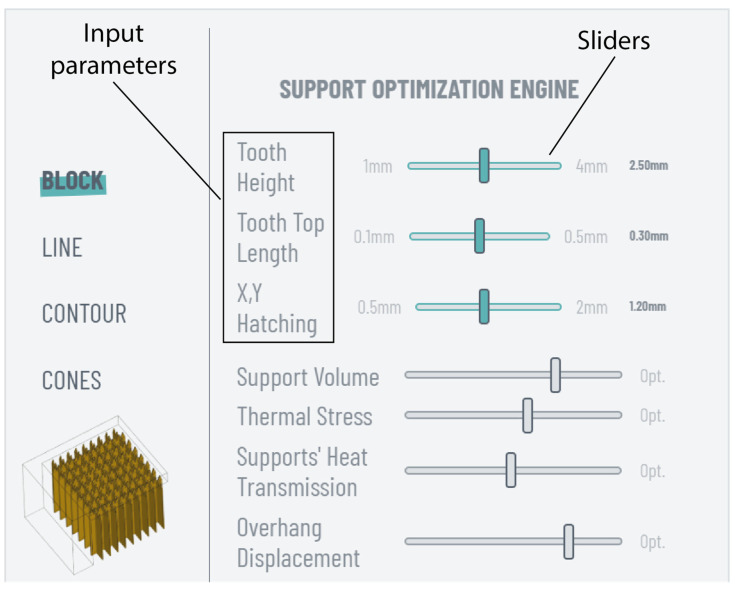
“Support optimisation engine” interface.

Concerning the support optimisation engine, this tool can be accessed in the top-right corner of the “settings” panel, represented by an open-book icon, as shown in Figure 5. With an interactive interface, it allows users to receive optimisation suggestions for block, line, contour, and cone support structures. The interface is illustrated in Figure 9. Utilising horizontal sliders, users can select and tune input parameters such as support density, tooth height, and tooth top length, receiving feedback on those parameters that better optimise support volume, thermal stress, supports’ heat transmission, and overhang displacement. This feedback is displayed on the four grey horizontal sliders beneath. Here, it should be restated that the optimisation results are exclusively based on the findings of the authors’ previously published work [14], where the thermal behaviour of the four mentioned support types, using L-shaped specimens made of titanium, was investigated. Subsequent experimentation and data collection are anticipated to enhance the functionality of the support optimisation engine, incorporating more input parameters and optimisation outputs such as support removability.

**Figure 10:**
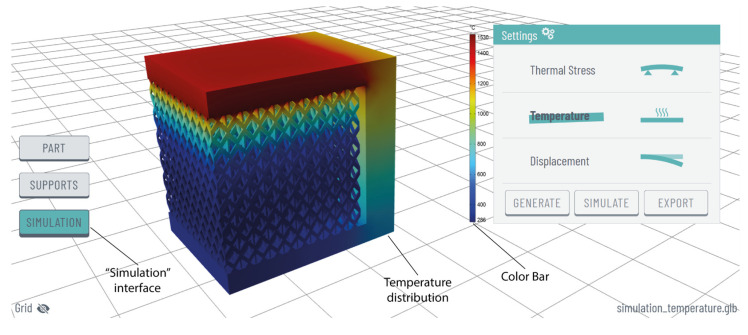
“Simulation” interface and temperature distribution numerical study.

Upon receiving optimisation suggestions for block, line, contour, and cone support structures, and subsequently generating block-type supports with diamond perforations, users can utilise the “simulate” option (see Figure 5) in the “settings” panel to conduct a comprehensive numerical study. This study, within the “simulation” interface, enables the examination of thermal stress, temperature distribution, and displacement on both the part and supports. Users have the flexibility to select their preferred display option through an interactive and user-friendly interface as illustrated in Figure 10. Currently, the simulation module operates in demonstration mode, simulating and visualising exclusively the illustrated 3D model as described in Section 2.5. Future iterations will integrate improved communication between the simulation software and the web platform, facilitated by a robust network infrastructure.

**Figure 11:**
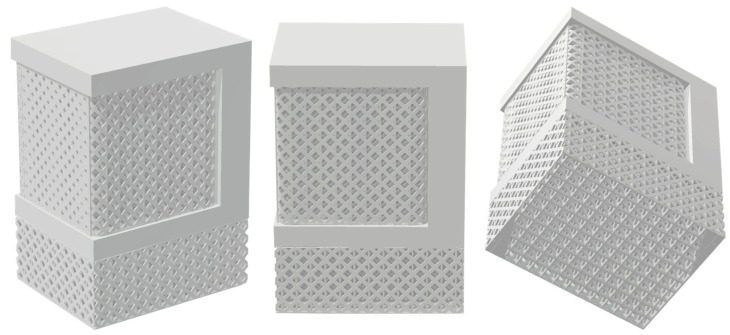
Solid support structures resulting from exporting the assembly via the web platform.

The final phase, following the generation and optimisation of support structures, involves exporting the prepared assembly. To visualise supports on the platform, non-solid geometries have been implemented; however, users have the flexibility to customise support thickness as needed through the “support thickness” option. This option, located at the bottom of the “settings” panel (see Figure 5), allows users to export solid supports (or non-solid if zero thickness is selected) in STL format. Figure 11 illustrates both the exported model and the generated support structures in STL format after applying a support thickness of 0.2 mm (default value). Generating solid supports within the platform is essential for conducting the thermomechanical simulations, as non-solid supports cannot undergo numerical analysis. Moreover, the exported solid assembly, including both the part and supports, can be utilised for additional analysis (e.g., static, fluid, etc.) or any pre-slicing procedures before the final print.

### 3.2. Online Access and Functionality Accuracy

Drawing from the user journey outlined in Section 3.1, the first release (v0.6) of our web-based support generation and optimisation platform is accessible online at no cost via https://slmsupportgeneration.web.app/ (accessed on 22 February 2024). Users can import 3D geometries of diverse sizes and complexities enabling the generation of block-type support structures with diamond perforations. Regarding the specimen used in Section 3.1, it is available in the Supplementary Materials, designated as ‘STL specimen_1’. Moreover, to ensure the accuracy of the functionality towards the input parameters, their numeric ranges, and the outcome, the following guidelines are proposed.

- Imported model: Import part only in STL format.
- Build plate volume: For this version, there is no specific volume limit; however, smaller parts are easier to handle, since they require less time for import and support generation.
- Orientation: Choose from 11 options tailored to user preferences.
- Surface angle: The default surface angle value is 50°. Tune accordingly, update, and generate.
- x, y Hatching: The default value is 2 mm, with suggested values ranging from 1 mm to 3 mm. Values lower than 1 mm may lead to geometric issues, while values greater than 3 mm may result in collapsed overhangs and are not recommended.
- Beam: The default value is 0.5 mm. Suggested values can range between 0.1 mm and 1 mm. Values greater than 1 mm may result in geometric issues.
- Angle: The default and suggested value is 45°. Other values higher or lower are still experimental and may result in incorrect support generation.
- Tooth height and tooth top length: These two options exist as input parameters, but this geometry will be developed in future iterations.
- Support thickness: The default value is 0.2 mm. Suggested values range from 0 mm (non-solid) to 0.5 mm. Upon applying a new value, generate first and then export. The thickness is currently not visualised on the platform interface, but will be visible upon export.
- Advanced settings: Can be adjusted based on the user’s expertise.
- Export: Both the part and support structures will be exported in STL format, incorporating the selected support thickness.
- Simulation: Currently available in demo mode and limited to a specific model—the one described in Section 3.1. This STL specimen can be downloaded from the Supplementary Materials titled ‘STL specimen_1’.
- Loading times: Importing and surface angle modification may take up to 45 s, while support generation typically requires up to 30 s. Deviations beyond these durations may result in dysfunctional behaviour.
- Troubleshooting: If loading persists for more than 1 min or support generation looks incorrect according to the selected parameters, return to the “part” interface to adjust the inputs and regenerate, or reload the page to import the model again. Alternatively, starting a new browser session may resolve the issue.

## 4. Conclusions

This study introduces the first release (v0.6) of a web-based platform designed for the generation and optimisation of support structures in metal AM and PBF-LB/M systems. In a user-friendly environment, users can import 3D files, orient components, generate support structures, access optimisation suggestions for various supports, conduct thermomechanical simulations, and finally export the finalised build job. Compared to other slicing and simulation software currently available on the market, this platform will offer distinct advantages. It will be accessible online at no cost, will be compatible with any web browser, and will accommodate not only experts in the field but also users less experienced with metal AM. Moreover, it will be suitable for use with a wide range of 3D printing machines, including both industrial and desktop models, as well as machines designed exclusively for research purposes.

Hence, through this platform, the AM industry stands to gain various benefits, including the adoption of PBF-LB/M as a production method, resulting in reduced printing times and post-processing, guaranteed successful prints with zero defects, minimised material usage for support structures, decreased instances of damaged parts during post-processing, and lowered CO_2_ laser emissions and printing costs.

In the upcoming stages, our focus will shift towards developing the second release of the web platform, where we will improve existing features and integrate new ones to enhance its capabilities. This endeavour aims to introduce comprehensive slicing software tailored for support generation and optimisation in metal AM and PBF-LB/M systems. Specifically, the orientation feature will be enhanced by prioritising the optimal solution at the top of the list, aiming to minimise the need for support material, reduce printing time, and lower overall costs. The geometry of the tooth area will be further developed, and the corresponding input parameters, such as tooth height and tooth top length, will be made available for support generation. Moreover, a network is set to be established, facilitating communication between the simulation software and the web platform via shared online servers. This setup will allow users to initiate and conduct thermomechanical simulations directly within the web platform, utilising their preferred 3D model.

## Data Availability

Data are contained within the article and Supplementary Materials.

## Supplementary Materials

The following supporting information can be downloaded at https://www.mdpi.com/article/10.3390/ma17071639/s1. Equations for support optimisation module, STL specimen_1, STL specimen_2.
