# Supplementary material for: An Interactive Web-Based Platform for Support Generation and Optimisation for Metal Laser Powder Bed Fusion
Source: Materials (Basel). 2024 Apr 3;17(7):1639. doi: 10.3390/ma17071639 (PMC11012501; doi:10.3390/ma17071639)
Supplement: Supplementary file 1 [file materials-17-01639-s001.zip › Equations for support optimization module.pdf]

# Equations for Support Optimization Module

## 1. Block-type Supports

**Support Volume** = 6991,79410 - 137,65809 Tooth Height +137,90833 Tooth Top Length - 5247,51284 X, Y Hatching + 71,08333 Tooth Height * Tooth Top Length + 43,15556 Tooth Height * X, Y Hatching - 117,00000 Tooth Top Length * X, Y Hatching - 0,004938 Tooth Height$^2$ - 0,277778 Tooth Top Length$^2$ + 1281,04691 X, Y Hatching$^2$

**Thermal Stress** = 6,42084e+09 + 2,17099e+07 Tooth Height + 1,33333e+07 Tooth Top Length - 2,44480e+09 X, Y Hatching + 1,41667e+08 Tooth Height * Tooth Top Length + 3,33333e+07 Tooth Height * X, Y Hatching - 1,66667e+07 Tooth Top Length * X, Y Hatching - 2,86420e+07 Tooth Height$^2$ + 2,63889e+08 Tooth Top Length$^2$ + 4,80988e+08 X, Y Hatching$^2$

**Supports' Heat Transmission** = 1127,30185 - 14,01019 Tooth Height + 87,58333 Tooth Top Length - 226,57593 X, Y Hatching + 22,91667 Tooth Height * Tooth Top Length - 0,555556 Tooth Height * X, Y Hatching + 2,50000 Tooth Top Length * X, Y Hatching - 0,074074 Tooth Height$^2$ - 141,66667 Tooth Top Length$^2$ + 40,59259 X, Y Hatching$^2$

**Overhang Displacement** = 0,450624 - 0,011220 Tooth Height + 0,035083 Tooth Top Length -0,036707 X, Y Hatching - 0,010417 Tooth Height * Tooth Top Length + 0,005222 Tooth Height * X, Y Hatching - 0,014167 Tooth Top Length * X, Y Hatching + 0,002123 Tooth Height$^2$ + 0,006944 Tooth Top Length$^2$ + 0,008494 X, Y Hatching$^2$

## 2. Line-type Supports

**Support Volume** = 3623,77256 - 69,47302 Tooth Height + 66,09167 Tooth Top Length - 2850,26494 Cross Line Interval + 36,91667 Tooth Height * Tooth Top Length + 21,02222 Tooth Height * Cross Line Interval - 57,00000 Tooth Top Length * Cross Line Interval - 0,017284 Tooth Height$^2$ + 0,277778 Tooth Top Length$^2$ + 741,61975 Cross Line Interval$^2$

**Thermal Stress** = 1,12473e+10 + 6,48272e+07 Tooth Height + 2,20667e+09 Tooth Top Length - 7,13657e+09 Cross Line Interval - 1,66667e+08 Tooth Height * Tooth Top Length + 6,44444e+07 Tooth Height * Cross Line Interval - 3,33333e+08 Tooth Top Length * Cross Line Interval - 2,98765e + 07 Tooth Height$^2$ - 1,55556e+09 Tooth Top Length$^2$ + 1,86272e+09 Cross Line Interval$^2$

**Supports' Heat Transmission** = 1103,19938 - 18,45586 Tooth Height + 96,16667 Tooth Top Length - 353,07840 Cross Line Interval + 14,58333 Tooth Height * Tooth Top Length + 2,55556 Tooth Height * Cross Line Interval - 19,16667 Tooth Top Length * Cross Line Interval + 0,617284 Tooth Height$^2$ - 90,27778 Tooth Top Length$^2$ + 98,46914 Cross Line Interval$^2$

**Overhang Displacement** = 0,480020 - 0,006101 Tooth Height + 0,007917 Tooth Top Length - 0,127547 Cross Line Interval  0,016667 Tooth Height * Tooth Top Length + 0,004444 Tooth Height * Cross Line Interval - 0,011667 Tooth Top Length * Cross Line Interval + 0,002469 Tooth Height$^2$ + 0,051389 Tooth Top Length$^2$ + 0,046321 Cross Line Interval$^2$

## 3. Contour-type Supports

**Support Volume** = 3545,32790 - 69,64716 Tooth Height + 66,00000 Tooth Top Length - 2661,23210 Contour Offset + 37,00000 Tooth Height * Tooth Top Length + 21,11111 Tooth Height * Contour Offset - 56,66667 Tooth Top Length * Contour Offset - 0,012346 Tooth Height$^2$ + 0,555556 Tooth Top Length$^2$ + 666,79506 Contour Offset$^2$

**Thermal Stress** = 6,47756e+09 - 2,08025e+06 Tooth Height + 6,43333e+08 Tooth Top Length - 3,03683e+09 Contour Offset + 9,16667e+07 Tooth Height * Tooth Top Length + 2,22222e+06 Tooth Height * Contour Offset - 1,83333e+08 Tooth Top Length * Contour Offset - 1,28395e+07 Tooth Height$^2$ - 7,22222e+08 Tooth Top Length$^2$ + 8,81975e+08 Contour Offset$^2$

**Supports' Heat Transmission** = 1137,57253 - 19,26790 Tooth Height + 125,25000 Tooth Top Length - 276,45802 Contour Offset + 20,00000 Tooth Height * Tooth Top Length + 1,55556 Tooth Height * Contour Offset - 31,66667 Tooth Top Length * Contour Offset + 0,691358 Tooth Height$^2$ - 111,11111 Tooth Top Length$^2$ + 56,98765 Contour Offset$^2$

**Overhang Displacement** = 0,437369 - 0,000096 Tooth Height - 0,015917 Tooth Top Length - 0,013870 Contour Offset - 0,018333 Tooth Height * Tooth Top Length + 0,002889 Tooth Height * Contour Offset - 0,005000 Tooth Top Length * Contour Offset + 0,002370 Tooth Height$^2$ + 0,070833 Tooth Top Length$^2$ + 0,000593 Contour Offset$^2$

## 4. Cones-type Supports

**Support Volume** = 777,36969 + 725,50333 Contact Platform + 3853,23333 Contact Part - 1597,69062 Spacing - 286,00000 Contact Platform * Contact Part - 93,00000 Contact Platform * Spacing - 561,33333 Contact Part * Spacing - 13,24444 Contact Platform$^2$ - 3076,11111 Contact Part$^2$ + 484,51358 Spacing$^2$

**Thermal Stress** = 1,27083e+11 - 9,02523e+10 Contact Platform - 3,92625e+11 Contact Part + 2,67620e+10 Spacing + 9,97750e+10 Contact Platform * Contact Part - 2,75233e+10 Contact Platform * Spacing - 8,01500e+10 Contact Part * Spacing + 2,75200e+10 Contact Platform$^2$ + 5,09500e+11 Contact Part$^2$ + 1,55733e+10 Spacing$^2$

**Supports' Heat Transmission** = 764,77870 - 45,93333 Contact Platform + 363,00000 Contact Part - 66,52593 Spacing - 17,50000 Contact Platform * Contact Part + 11,66667 Contact Platform * Spacing - 155,00000 Contact Part * Spacing + 11,33333 Contact Platform$^2$ + 83,33333 Contact Part$^2$ + 23,70370 Spacing$^2$

**Overhang Displacement** = 0,496003 - 0,024433 Contact Platform + 0,030333 Contact Part - 0,027511 Spacing + 0,002500 Contact Platform * Contact Part + 0,001667 Contact Platform * Spacing - 0,001667 Contact Part * Spacing + 0,004000 Contact Platform$^2$ + 8,25825e-16 Contact Part$^2$ + 0,007111 Spacing$^2$
